# Association between novel anthropometric indices and overactive bladder: a population-based study

**DOI:** 10.3389/fnut.2025.1493792

**Published:** 2025-01-22

**Authors:** Heng Liu, Huqiang Dong, Mingchu Jin, Yu Zhou, Haidong Hao, Yutang Yuan, Hongtao Jia, Min He

**Affiliations:** ^1^Department of Urology, Renmin Hospital, Hubei University of Medicine, Shiyan, China; ^2^School of Public Health, Ningxia Medical University, Yinchuan, China; ^3^Department of Respiratory and Critical Care Medicine, Renmin Hospital, Hubei University of Medicine, Shiyan, China

**Keywords:** overactive bladder, WWI, BRI, RFM, ABSI, NHANES

## Abstract

**Background:**

Abdominal obesity is recognized as a key risk factor for developing OAB. However, traditional measures of obesity, such as the waist-to-height ratio (WHtR), waist circumference, and body mass index (BMI), may not sufficiently capture fat distribution in the body. This study aims to evaluate the relationship between novel anthropometric indices and OAB, providing a more accurate assessment of obesity-related risk factors.

**Methods:**

The National Health and Nutrition Examination Survey (NHANES) data from 2007 to 2018 were utilized, comprising 27,560 participants. To assess the association and discriminative ability of novel anthropometric indices, including the Body Roundness Index (BRI), A Body Shape Index (ABSI), Waist-to-Weight Index (WWI), and Relative Fat Mass (RFM), with OAB, we employed multivariable logistic regression, restricted cubic spline (RCS) analysis, subgroup analysis, and receiver operating characteristic (ROC) curve methods.

**Results:**

Multivariable logistic regression analysis indicated that higher levels of novel anthropometric indices were positively associated with OAB prevalence. One z-score increase in WWI, BRI, RFM, and ABSI was associated with a 16, 31, 57, and 5% higher likelihood of OAB, respectively. RCS analysis revealed a non-linear relationship between RFM and OAB. ROC analysis indicated that WWI (AUC = 0.680) and RFM (AUC = 0.661) provided better diagnostic accuracy than traditional measures such as BMI (AUC = 0.599). Subgroup analyses supported the robustness of these findings.

**Conclusion:**

Novel anthropometric indices were positively associated with OAB prevalence. WWI and RFM demonstrated significantly better diagnostic value for OAB than BMI and WHtR. Future studies should investigate the potential of combining multiple anthropometric indices to improve predictive accuracy and conduct prospective studies to determine causality.

## 1 Background

Overactive bladder (OAB) is characterized by an abrupt and intense need to urinate, often accompanied by nocturia, urgency incontinence, and frequent urination. It reflects lower urinary storage dysfunction without underlying pathological conditions, such as urinary tract infections (1). OAB affects more and more adult males in the United States than 14% of them, according to studies (2–4). The etiology of OAB is complex, with known risk factors such as urinary tract infections, anxiety, circadian rhythm disorders, and other contributing factors (5–7). OAB has a significant adverse impact on patients’ quality of life, affecting mental health and sleep, while also placing a heavy burden on the healthcare system (8, 9). Research indicates that healthcare costs for individuals with overactive bladder (OAB) are approximately 2.5 times higher than for those without the condition (8). As the population ages, OAB has emerged as a growing and significant public health concern.

Abdominal obesity increases intra-abdominal pressure, which may lead to the development of stress urinary incontinence (SUI) as well as OAB (3, 10). The waist circumference (WC), body mass index (BMI), and waist-to-height ratio (WHtR) are conventional measures of obesity (11). However, these metrics cannot effectively differentiate between subcutaneous and visceral fat, both of which are strongly associated with the prevalence of OAB (3). Although novel measurement methods, such as magnetic resonance imaging, can more accurately assess fat distribution, their high cost and technical complexity limit their widespread application (12, 13). Therefore, it has been shown that several recently established and more practical measuring indices, including the relative fat mass (RFM), weight-adjusted waist index (WWI), body shape index (ABSI), and body roundness index (BRI), are reliable indicators of visceral fat (14–16). Among them, RFM is strongly associated with mortality, depression, dyslipidemia, and metabolic syndrome, while ABSI and BRI are effective in predicting percent body fat and visceral fat (17–19). According to Zhang et al., the risk of OAB correlates with several of these novel measures (20). These findings are important for the accurate identification of high-risk individuals as well as the development of early interventions.

It is important to do a thorough investigation and assessment of these measurements since it is currently unknown which anthropometric parameters are best for screening OAB. In order to predict the risk of OAB in adult U.S. citizens, this study aimed to evaluate and contrast the validity of many anthropometric indices, such as BMI, WHTR, ABSI, BRI, WWI, and RFM. This study leverages a large dataset from NHANES, providing an adequate sample size and diverse demographic information. By conducting this research, we aim to identify the most effective predictors of OAB risk, thereby providing a scientific basis for clinical practice and public health policy to promote the prevention of urologic diseases.

## 2 Materials and methods

### 2.1 Data statement

The National Health and Nutrition Examination Survey (NHANES), conducted by the National Center for Health Statistics (NCHS) with Ethics Committee approval, involves five core components: demographic data, physiological assessments, laboratory tests, health questionnaires, and nutritional surveys. Participants provide written informed consent. The database is available to researchers, facilitating numerous studies and informing health policy development.

### 2.2 Study population

**Figure 1:**
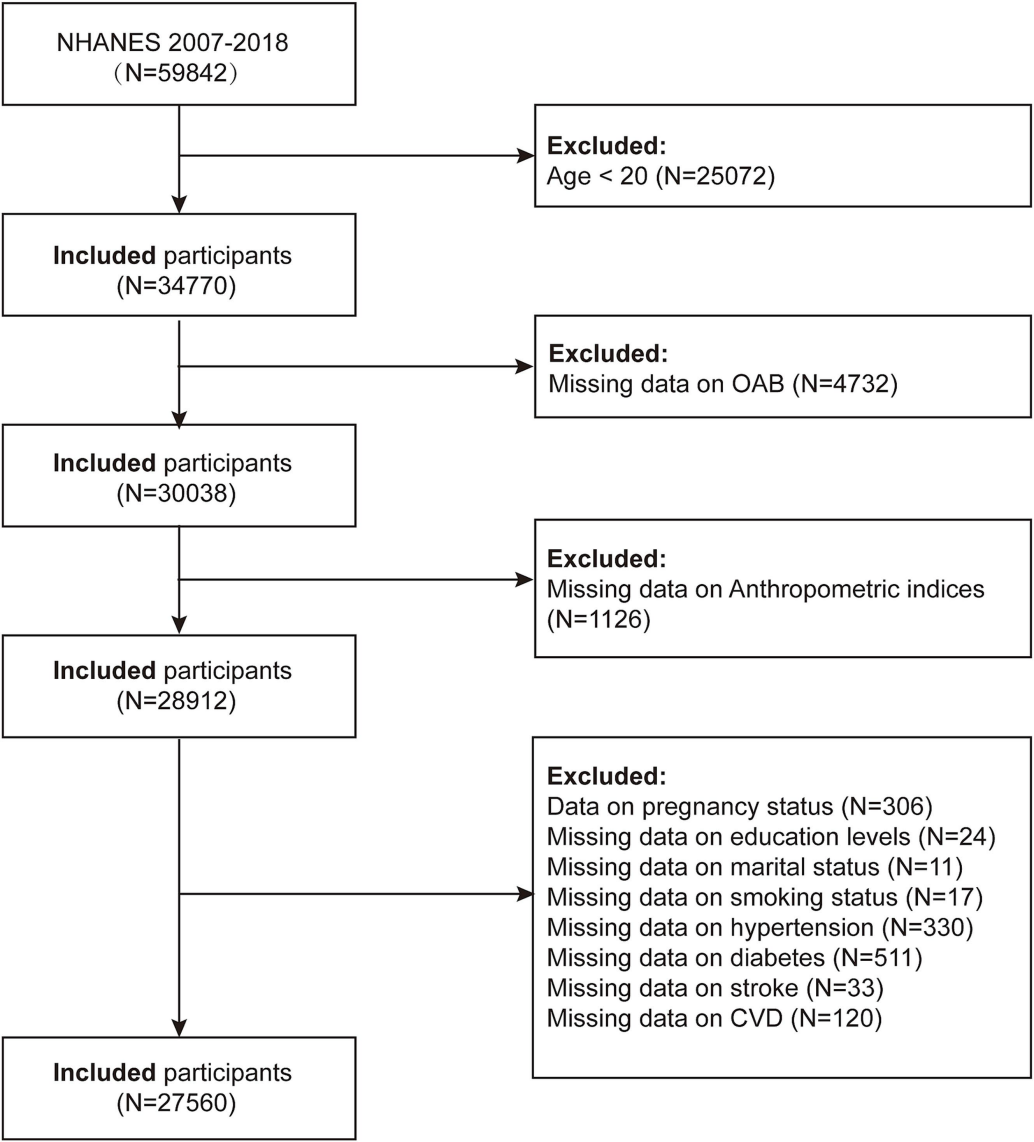
Study population flow chart 2007–2018.

Using data from 2007 to 2018, our research aimed to assess the association between novel anthropometric indices and the prevalence of OAB, as well as to explore the predictive power of these indices for OAB. Initially, 59,842 participants were included. The exclusion criteria were as follows: (1) Individuals who are under 20 years old (*n* = 25,072); (2) Participants with missing OAB data (*n* = 4,732); (3) Participants with missing novel anthropometric index data (*n* = 1,126); (4) Participants with pregnancy status (*n* = 306); (5) Participants with missing education level data (*n* = 24); (6) Participants with missing marital status data (n = 11); (7) Participants with missing smoking status data (*n* = 17); (8) Participants with missing hypertension data (*n* = 330); (9) Participants with missing diabetes data (*n* = 511); (10) Participants with missing stroke data (*n* = 33, 11) Participants with other missing data (*n* = 120). A total of 27,650 participants were included in the final analyses. (Figure 1).

### 2.3 Assessment of novel anthropometric indices

The NHANES database collects comprehensive health data, including body measurements and self-reported information. At mobile screening centers, trained health technicians measure weight, height, and waist circumference using standardized methods outlined in the procedure manual. Each measurement involves both a health technician and an accompanying recorder. These variables were subsequently used to calculate key anthropometric indices, including BMI, WHtR, WWI, BRI, RFM, and ABSI (21, 22).


$$BMI=\frac{\mathrm{Weight}\;\left(\mathrm{kg}\right)}{\mathrm{Heigh}t^{2}\;\left(m\right)}$$

$$\mathrm{WHtR}=\frac{WC\;\left(\mathrm{cm}\right)}{\mathrm{Height}\;\left(\mathrm{cm}\right)}$$

$$WWI=\frac{WC\;\left(\mathrm{cm}\right)}{\sqrt{\mathrm{Weight}\;\left(\mathrm{kg}\right)}}$$

$$BRI=364.2-365.5\times \sqrt{1-{\left(\frac{WC\;\left(m\right)}{2\pi \times 0.5\times \mathrm{Height}\;\left(m\right)}\right)}^{2}}$$

$$For\;\mathrm{men}:RFM=64-\left(20\times \frac{\mathrm{Height}\;\left(\mathrm{cm}\right)}{WC\;\left(\mathrm{cm}\right)}\right)$$

$$For\;\mathrm{women}:RFM=76-\left(20\times \frac{\mathrm{Height}\;\left(\mathrm{cm}\right)}{WC\;\left(\mathrm{cm}\right)}\right)$$

$$\mathrm{ABSI}=\frac{WC\;\left(m\right)}{\left(BMI^{\frac{2}{3}}\times \mathrm{Heigh}t^{\frac{1}{2}}\left(m\right)\right)}$$

### 2.4 Assessment of overactive bladder

Urinary urgency, characterized by a sudden and difficult-to-delay need to urinate, is the hallmark symptom of overactive bladder (OAB), often accompanied by frequent urination, nocturia, and urge incontinence. The NHANES database employs the Renal Condition - Urologic Questionnaire to evaluate symptoms associated with OAB, serving as a key resource for assessing this condition. Specifically, participants responded to questions such as, “In the past 12 months, have you leaked urine or lost control of urination when you had the urge or pressure to urinate and were unable to go to the toilet quickly?” and “How often does this happen?” These questions are used to assess the presence and frequency of OAB symptoms. To measure nocturia severity, participants were asked, “Over the past 30 days, from the time you went to bed at night until the time you woke up in the morning, what was the most typical number of times you woke up to urinate each night?” The severity of OAB was further evaluated using a simple Overactive Bladder Symptom Score (OABSS) (23). Participants with a total score of 3 or higher were defined in this study as having overactive bladder (24). See Supplementary Table S1 for a detailed explanation of the scoring methodology.

### 2.5 Assessment of covariates

In this study, covariates included gender (male/female), age, and race (Mexican American, Other Hispanic, Non-Hispanic White, Non-Hispanic Black, and Other Race). Education level was categorized as less than high school, high school, and greater than high school. Marital status was classified as never married, married/living with a partner, and widowed/divorced/separated. The family income poverty ratio (PIR) was divided into three groups: <1.3 (low income), 1.3–3.5 (moderate income), and > 3.5 (high income). Body mass index (BMI) was categorized into three groups: <25 kg/m^2^, 25–30 kg/m^2^, and ≥ 30 kg/m^2^. Smoking status was classified as a current smoker (≥100 cigarettes and currently smokes), ex-smoker (≥100 cigarettes but no longer smokes), and never-smoker (≤100 cigarettes or never smoked). An alcohol drinker was defined as someone who had consumed at least 12 alcoholic beverages in any one year of their life. Diabetes was defined as either a physician diagnosis or a fasting blood glucose level of ≥126 mg/dL. Hypertension was defined as either a previous diagnosis, current use of antihypertensive medication, or an average of three blood pressure readings ≥140/90 mmHg. Stroke was classified as yes or no. Cardiovascular disease was classified as yes or no. In our study, covariates were selected based on their established associations in previous studies and their potential influence on exposure (novel anthropometric indices) and outcome (OAB prevalence).

### 2.6 Statistical analysis

The statistical analyses followed the data analysis guidelines provided by NHANES, using appropriate NHANES weights to account for the complex multistage cluster sampling design. For continuous variables, we reported the survey-weighted mean (95% CI), and for categorical variables, the survey-weighted percentage (95% CI). Weighted linear regression or weighted chi-square tests were used to assess differences between the OAB and non-OAB groups. In this study, all anthropometric variables were z-score transformed as follows: z score = (index - index_mean) /index_sd.

Three weighted logistic regression models were used in this study to explore the correlation between novel anthropometric indices (WWI, BRI, RFM, ABSI) and OAB. Model 1 was unadjusted. Model 2 was adjusted for age, gender, and race. Model 3 was further adjusted to include education level, marital status, BMI, PIR, smoking, alcohol consumption, diabetes, hypertension, cardiovascular disease, and stroke, in addition to the covariates in Model 2. The study then tested for nonlinear associations between novel anthropometric indices and OAB using restricted cubic splines (RCS), after adjusting for covariates. Subgroup analyses and interaction tests were conducted on potential confounders listed in the baseline table to explore variations in associations between subgroups. Finally, receiver operating characteristic (ROC) curves were used to evaluate the predictive power of novel anthropometric indices (WWI, BRI, RFM, ABSI, WHtR, BMI) for OAB, and the area under the curve (AUC) values were compared. Statistical analyses were performed using R (http://www.r-project.org) and EmpowerStats (http://www.empowerstats.com), with significance set at *p* < 0.05.

## 3 Results

### 3.1 Baseline characteristics of the study population

A total of 27,560 participants were included in this study, and the weighted prevalence of OAB was 20.12% (19.30, 20.97%). Compared with non-OAB participants, OAB patients had significantly higher levels of ABSI z-score, BRI z-score, WWI z-score, and RFM z-score, which were 0.24 (0.20, 0.29), 0.35 (0.31, 0.38), 0.40 (0.36, 0.44), and 0.46 (0.43, 0.50), respectively, with significant differences between groups (*p* < 0.01). Additionally, the OAB group had a higher proportion of female patients, individuals who were widowed/divorced/separated, those with BMI ≥ 30, smokers, and participants with hypertension, diabetes, cardiovascular disease, or stroke, with significant differences between groups (*p* < 0.01). (Table 1).

**Table 1:** Weighted baseline characteristics of the study population.

Characteristics	Total (*n* = 27,560)	Non-OAB (*n* = 21,009)	OAB (*n* = 6,551)	*p*-value
Age, years, mean(95%CI)	47.54 (47.06, 48.01)	44.99 (44.50, 45.48)	57.65 (57.02, 58.29)	<0.0001
Gender, %(95%CI)				<0.0001
Male	49.62 (49.00, 50.25)	53.83 (53.04, 54.62)	32.91 (31.50, 34.36)	
Female	50.38 (49.75, 51.00)	46.17 (45.38, 46.96)	67.09 (65.64, 68.50)	
Race, % (95%CI)				<0.0001
Mexican American	8.42 (7.04, 10.05)	8.80 (7.36, 10.49)	6.91 (5.64, 8.45)	
Other Hispanic	5.75 (4.86, 6.79)	5.91 (4.97, 7.02)	5.10 (4.29, 6.06)	
Non-Hispanic White	67.46 (64.64, 70.16)	67.21 (64.38, 69.91)	68.46 (65.22, 71.53)	
Non-Hispanic Black	10.86 (9.52, 12.36)	10.07 (8.82, 11.47)	14.02 (12.10, 16.18)	
Other Race	7.51 (6.73, 8.37)	8.01 (7.19, 8.91)	5.51 (4.62, 6.57)	
Education level, % (95%CI)				<0.0001
Less than high school	15.09 (13.94, 16.31)	13.44 (12.35, 14.62)	21.61 (19.99, 23.33)	
High school	23.16 (22.09, 24.27)	22.64 (21.51, 23.82)	25.23 (23.49, 27.06)	
More than high school	61.75 (59.87, 63.59)	63.91 (61.99, 65.79)	53.16 (50.76, 55.54)	
Marital status, % (95%CI)				<0.0001
Never married	18.52 (17.35, 19.74)	20.36 (19.12, 21.66)	11.19 (9.99, 12.53)	
Married/Living with partner	63.33 (62.01, 64.62)	64.17 (62.83, 65.48)	60.00 (57.93, 62.03)	
Widowed/divorced/separated	18.16 (17.45, 18.89)	15.47 (14.80, 16.17)	28.81 (27.30, 30.37)	
PIR, % (95%CI)				<0.0001
< 1.3	21.99 (20.63, 23.40)	20.66 (19.35, 22.04)	27.24 (25.04, 29.57)	
1.3–3.5	34.61 (33.39, 35.84)	33.86 (32.54, 35.21)	37.56 (35.85, 39.29)	
≥ 3.5	43.41 (41.47, 45.37)	45.47 (43.51, 47.45)	35.20 (32.59, 37.91)	
BMI, % (95%CI)				<0.0001
< 25	29.49 (28.41, 30.60)	31.63 (30.42, 32.87)	20.98 (19.73, 22.30)	
25–30	33.01 (32.08, 33.95)	33.77 (32.74, 34.82)	29.96 (28.15, 31.84)	
≥ 30	37.50 (36.35, 38.68)	34.60 (33.33, 35.88)	49.05 (47.23, 50.88)	
Smoking status, % (95%CI)				<0.0001
Never	55.25 (54.02, 56.48)	56.71 (55.40, 58.01)	49.46 (47.33, 51.60)	
Now	19.78 (18.84, 20.75)	19.49 (18.52, 20.50)	20.92 (19.23, 22.72)	
Former	24.97 (24.04, 25.92)	23.80 (22.74, 24.89)	29.62 (27.92, 31.37)	
Alcohol intake, % (95%CI)				<0.0001
No	10.18 (9.38, 11.03)	9.11 (8.29, 10.00)	14.42 (13.20, 15.74)	
Yes	89.82 (88.97, 90.62)	90.89 (90.00, 91.71)	85.58 (84.26, 86.80)	
Hypertension, % (95%CI)				<0.0001
No	61.86 (60.73, 62.99)	67.00 (65.83, 68.16)	41.45 (39.90, 43.02)	
Yes	38.14 (37.01, 39.27)	33.00 (31.84, 34.17)	58.55 (56.98, 60.10)	
Diabetes, % (95%CI)				<0.0001
No	86.76 (86.14, 87.36)	89.71 (89.10, 90.28)	75.06 (73.62, 76.45)	
Yes	13.24 (12.64, 13.86)	10.29 (9.72, 10.90)	24.94 (23.55, 26.38)	
Stroke, %(95%CI)				<0.0001
No	97.35 (97.10, 97.57)	98.21 (98.00, 98.40)	93.92 (93.06, 94.68)	
Yes	2.65 (2.43, 2.90)	1.79 (1.60, 2.00)	6.08 (5.32, 6.94)	
CVD, % (95%CI)				<0.0001
No	93.39 (92.93, 93.83)	95.19 (94.74, 95.61)	86.25 (84.96, 87.44)	
Yes	6.61 (6.17, 7.07)	4.81 (4.39, 5.26)	13.75 (12.56, 15.04)	
WHtR, mean (95%CI)	0.59 (0.59, 0.59)	0.58 (0.58, 0.58)	0.63 (0.63, 0.64)	<0.0001
Weight, cm, mean (95%CI)	82.96 (82.47, 83.46)	82.49 (81.96, 83.03)	84.83 (83.93, 85.72)	<0.0001
Height, cm, mean (95%CI)	168.76 (168.55, 168.97)	169.67 (169.44, 169.90)	165.14 (164.76, 165.52)	<0.0001
WC, cm, mean (95%CI)	99.44 (98.99, 99.89)	98.19 (97.72, 98.67)	104.39 (103.70, 105.08)	<0.0001
ABSI, mean (95%CI)	0.08 (0.08, 0.08)	0.08 (0.08, 0.08)	0.08 (0.08, 0.08)	<0.0001
BRI, mean (95%CI)	5.30 (5.24, 5.36)	5.08 (5.02, 5.14)	6.17 (6.10, 6.25)	<0.0001
WWI, mean (95%CI)	10.97 (10.95, 11.00)	10.86 (10.84, 10.89)	11.40 (11.36, 11.43)	<0.0001
RFM, mean (95%CI)	35.25 (35.06, 35.43)	34.15 (33.96, 34.33)	39.60 (39.30, 39.90)	<0.0001
ABSI z-score, mean (95%CI)	−0.05 (−0.08, −0.03)	−0.13 (−0.15, −0.10)	0.24 (0.20, 0.29)	<0.0001
BRI z-score, mean (95%CI)	−0.06 (−0.09, −0.03)	−0.16 (−0.19, −0.14)	0.35 (0.31, 0.38)	<0.0001
WWI z-score, mean (95%CI)	−0.11 (−0.14, −0.08)	−0.24 (−0.26, −0.21)	0.40 (0.36, 0.44)	<0.0001
RFM z-score, mean (95%CI)	−0.04 (−0.06, −0.01)	−0.16 (−0.18, −0.14)	0.46 (0.43, 0.50)	<0.0001

For continuous variables: survey-weighted mean (95% CI), *P*-value was by survey-weighted linear regression (svyglm). For categorical variables: survey-weighted percentage (95% CI), *P*-value was by survey-weighted Chi-square test (svytable).

PIR, the ratio of income to poverty; BMI, body mass index; CVD, cardiovascular; WC, waist circumference; WHtR, waist-to-height ratio; WWI, weight-adjusted waist index; BRI, body roundness index; RFM, relative fat mass; ABSI, a body shape index.

### 3.2 Association between novel anthropometric indices and OAB

**Figure 2:**
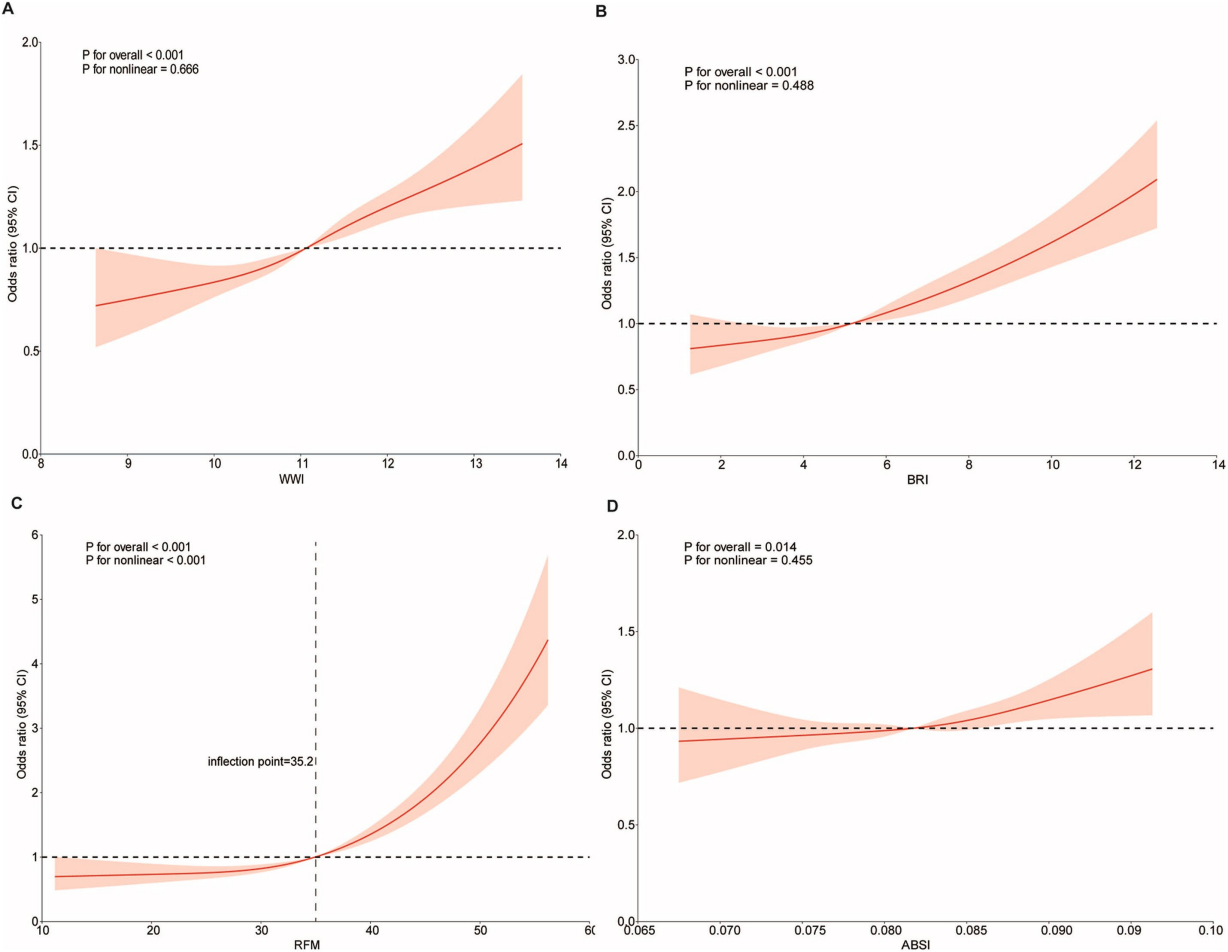
Dose-effect relationship between novel anthropometric indices and OAB. Age, gender, race, education level, marital status, PIR, BMI, smoking status, alcohol consumption, diabetes, hypertension, CVD, and stroke were adjusted. **(A)** WWI and OAB; **(B)** BRI and OAB; **(C)** RFM and OAB; **(D)** ABSI and OAB.

Table 2 presents the results of a multivariate logistic regression analysis examining the relationship between four novel anthropometric indices and OAB, revealing a positive correlation. In Model 3, each one-unit increase in the z-score of WWI was associated with a 16% higher likelihood of developing OAB (OR = 1.16, 95% CI: 1.11, 1.21). Similarly, for each one-unit increase in the z-score of BRI, the likelihood increased by 31% (OR = 1.31, 95% CI: 1.25, 1.37), while for RFM, the likelihood increased by 57% (OR = 1.57, 95% CI: 1.42, 1.73). For ABSI, a one-unit increase in z-score was linked to a 5% increase in the likelihood of developing OAB (OR = 1.05, 95% CI: 1.01, 1.09). Additionally, the four novel anthropometric indices were transformed from continuous variables into categorical variables (quartiles). In Model 3, participants in the highest quartile (Q4) were significantly more likely to develop OAB compared to those in the lowest quartile (Q1) (p for trend <0.05) (Table 2). Furthermore, the RCS analysis revealed no nonlinear associations between WWI, BRI, or ABSI and OAB (p for nonlinearity >0.05). Interestingly, RFM displayed an L-shaped nonlinear relationship with OAB, with a threshold of 35.2 (p for nonlinearity <0.05) (Figure 2).

**Table 2:** Weighted multivariate logistic regression between four novel anthropometric indices and OAB.

Characteristic	Model 1		Model 2		Model 3	
	OR (95% CI)	*p* value	OR (95% CI)	*P* value	OR (95% CI)	*P* value
WWI z-score						
Continuous	1.96 (1.90, 2.02)	<0.001	1.44 (1.39, 1.49)	<0.001	1.16 (1.11, 1.21)	<0.001
Q1	Reference		Reference		Reference	
Q2	1.87 (1.69, 2.06)	<0.001	1.36 (1.23, 1.51)	<0.001	1.15 (1.03, 1.28)	0.011
Q3	3.05 (2.77, 3.34)	<0.001	1.72 (1.55, 1.90)	<0.001	1.24 (1.11, 1.39)	0.001
Q4	5.66 (5.17, 6.19)	<0.001	2.47 (2.23, 2.74)	<0.001	1.44 (1.28, 1.62)	<0.001
*P* for trend		<0.001		<0.001		<0.001
BRI z-score						
Continuous	1.65 (1.61, 1.70)	<0.001	1.45 (1.41, 1.50)	<0.001	1.31 (1.25, 1.37)	<0.001
Q1	Reference		Reference		Reference	
Q2	1.54 (1.40, 1.69)	<0.001	1.11 (1.01, 1.23)	0.033	1.08 (0.96, 1.21)	0.217
Q3	2.41 (2.21, 2.63)	<0.001	1.49 (1.35, 1.64)	<0.001	1.27 (1.10, 1.47)	0.001
Q4	4.12 (3.78, 4.49)	<0.001	2.44 (2.22, 2.67)	<0.001	1.72 (1.46, 2.04)	<0.001
*P* for trend		<0.001		<0.001		<0.001
RFM z-score						
Continuous	1.81 (1.76, 1.87)	<0.001	1.84 (1.75, 1.95)	<0.001	1.57 (1.42, 1.73)	<0.001
Q1	Reference		Reference		Reference	
Q2	1.60 (1.46, 1.75)	<0.001	1.27 (1.15, 1.40)	<0.001	1.06 (0.94, 1.19)	0.349
Q3	2.12 (1.94, 2.32)	<0.001	1.98 (1.75, 2.23)	<0.001	1.40 (1.20, 1.64)	<0.001
Q4	4.64 (4.26, 5.06)	<0.001	4.02 (3.50, 4.62)	<0.001	2.14 (1.71, 2.67)	<0.001
*P* for trend		<0.001		<0.001		<0.001
ABSI z-score						
Continuous	1.50 (1.46, 1.55)	<0.001	1.14 (1.10, 1.18)	<0.001	1.05 (1.01, 1.09)	0.008
Q1	Reference		Reference		Reference	
Q2	1.21 (1.11, 1.32)	<0.001	1.00 (0.91, 1.09)	0.926	0.93 (0.85, 1.03)	0.161
Q3	1.66 (1.52, 1.80)	<0.001	1.12 (1.02, 1.23)	0.017	0.99 (0.90, 1.09)	0.828
Q4	2.72 (2.51, 2.95)	<0.001	1.34 (1.22, 1.48)	<0.001	1.08 (0.98, 1.20)	0.115
*P* for trend		<0.001		<0.001		0.028

Model 1: No covariates were adjusted.

Model 2: Adjusted for age, gender, and race.

Model 3: Adjusted for age, gender, race, education level, marital status, PIR, BMI, smoking status, alcohol consumption, diabetes, hypertension, CVD, and stroke.

### 3.3 Subgroup analysis

**Figure 3:**
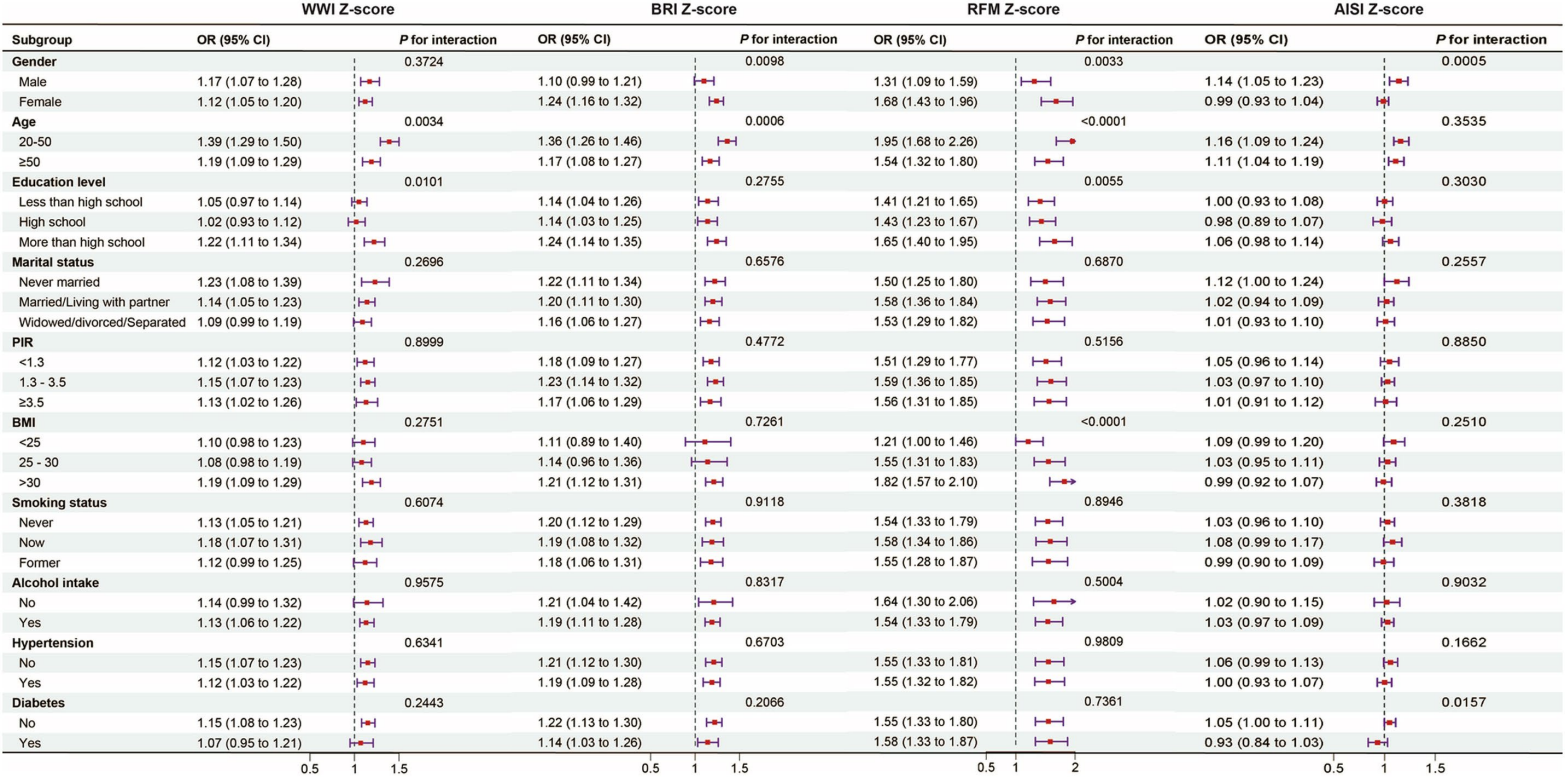
Subgroup analysis and interaction test between novel anthropometric indices and OAB. Note 1: The above model was adjusted for gender, age, race, education level, marital status, PIR, BMI, smoking status, alcohol consumption, diabetes, hypertension, CVD, and stroke. Note 2: In each case, the model was not adjusted for the stratification variable.

The results of the study showed positive associations between the four novel anthropometric indices and OAB prevalence across all subgroups. Interestingly, the positive association between WWI and OAB prevalence was stronger among participants aged 20–50 years (per z-score, OR = 1.39, 95% CI: 1.29, 1.50) and those with education beyond high school (per z-score, OR = 1.22, 95% CI: 1.11, 1.34) (*P* for interaction <0.05). The positive association between BRI and OAB prevalence was stronger in women (per z-score, OR = 1.24, 95% CI: 1.16, 1.32) and individuals aged 20–50 years (per z-score, OR = 1.36, 95% CI: 1.26, 1.46) (*P* for interaction <0.05). The positive association between RFM and OAB prevalence was stronger in women (per z-score, OR = 1.68, 95% CI: 1.43, 1.96), in individuals aged 20–50 years (per z-score, OR = 1.95, 95% CI: 1.68, 2.26), and in those with an education level beyond high school (per z-score, OR = 1.65, 95% CI: 1.40, 1.95) (*P* for interaction <0.05). The positive association between ABSI and OAB prevalence was stronger in men (per z-score, OR = 1.14, 95% CI: 1.05, 1.23) (*P* for interaction <0.05). (Figure 3).

### 3.4 ROC analysis

**Figure 4:**
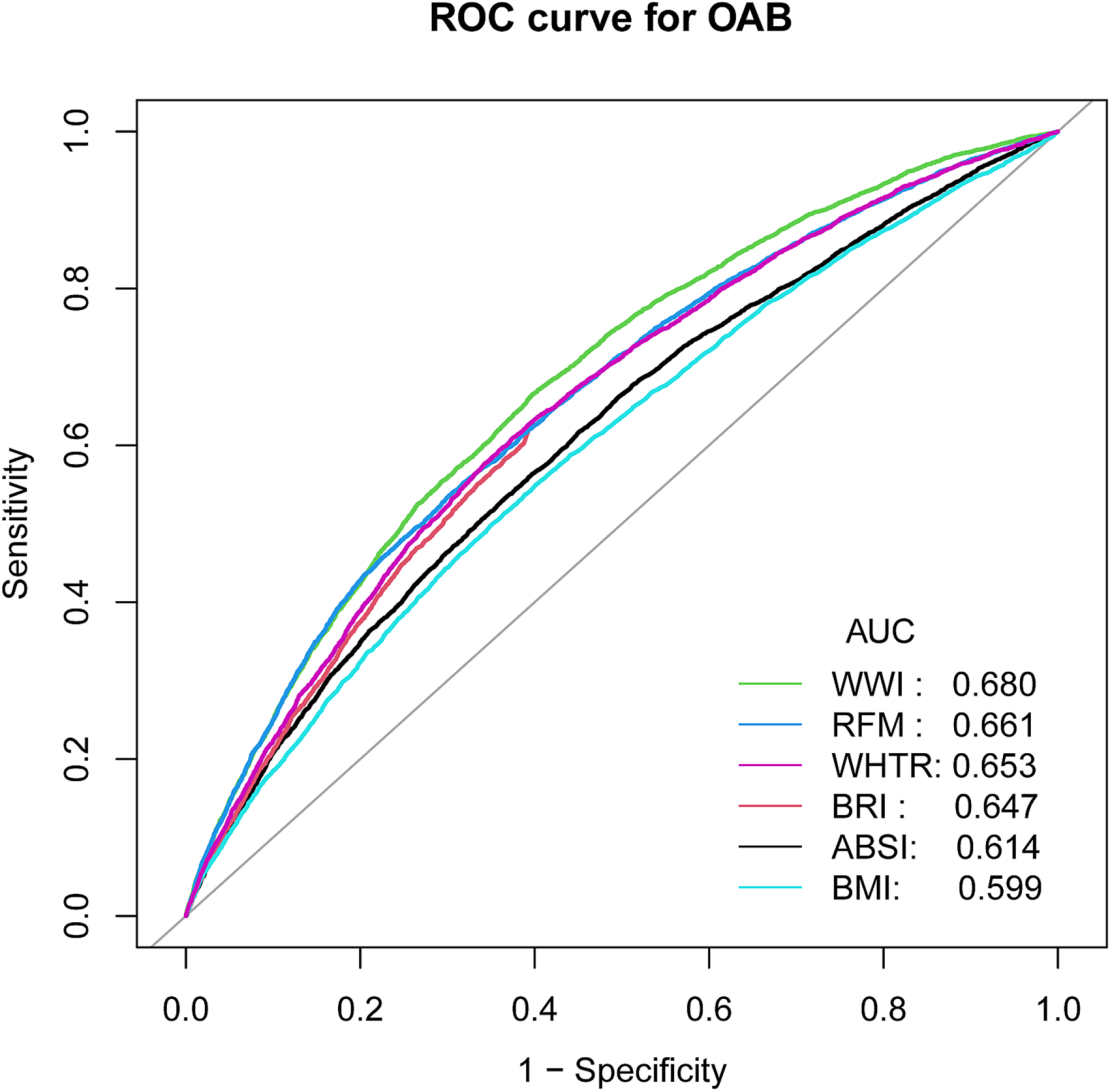
ROC curves and the AUC values of the anthropometric indicators (WWI, BRI, RFM, ABSI, WHTR, BMI) in diagnosing OAB.

In this study, the ability of six anthropometric indices to identify patients with OAB was assessed using ROC curves. The ROC curve analysis demonstrated that WWI and RFM were significantly more diagnostic of OAB than WHtR, BRI, BMI, and ABSI. The areas under the curves (AUC) for WWI, RFM, WHtR, BRI, ABSI, and BMI were 0.680, 0.661, 0.653, 0.647, 0.614, and 0.599, respectively. These findings suggest that WWI may have higher discriminatory ability and accuracy than other anthropometric indices (RFM, WHtR, BRI, ABSI, and BMI) in predicting the risk of OAB. (Figure 4) (Table 3).

**Table 3:** Comparison of AUC values for six anthropometric indicators.

Test	^a^AUC	95%CI^b^ low	95%CI upp	Best threshold	Specificity	Sensitivity	*P* for different in AUC
WHtR	0.6526	0.6450	0.6601	0.6054	0.6246	0.6100	Reference
BMI	0.5985	0.5906	0.6065	29.285	0.6112	0.5373	<0.001
ABSI	0.6144	0.6065	0.6222	0.0829	0.6373	0.5295	<0.001
BRI	0.6473	0.6398	0.6549	5.4305	0.6039	0.6295	1.000
WWI	0.6797	0.6724	0.6870	11.1482	0.6024	0.6652	<0.001
RFM	0.6607	0.6532	0.6683	39.2789	0.6941	0.5401	0.006

^aAUC, area under the curve.^

^b95%CI, 95% confidence interval.^

## 4 Discussion

Using the NHANES database, this study evaluates the diagnostic validity of four novel anthropometric indices for OAB prevalence, offering the first thorough investigation of the association between OAB and new anthropometric indicators. It offers a new perspective on OAB risk assessment, with findings indicating that individuals at high risk of OAB typically have elevated mean anthropometric values. In the fully adjusted model, all anthropometric indices were significantly correlated with OAB risk, and ROC curve analyses identified WWI as the most effective diagnostic indicator of OAB prevalence.

Studies have demonstrated that when it comes to predicting diabetes and cardiovascular illness, the WHtR is superior to BMI (25). Compared to conventional measurements, the BRI is a more accurate estimator of visceral fat index and body fat percentage (26). A recent study also demonstrated a correlation between elevated BRI and an increased incidence of overactive bladder (OAB) (20). Relative fat mass (RFM) is superior to BMI in predicting dyslipidemia and metabolic syndrome (18). Furthermore, studies have shown a correlation between RFM and mortality from every cause, with a threshold RFM value of 40% in women useful for diagnosing obesity and identifying those who are at a higher risk of passing away (17). WWI is a new index for measuring obesity that, when paired with standardized weight measurements, provides a more logical and straightforward method than BMI. Consistent with the results of this investigation, a recent study discovered that a greater WWI was linked to an increased incidence of OAB (27). This study further suggests that WWI has a more significant impact on OAB incidence than other markers of central obesity and serves as a more reliable predictive tool for gallstone risk.

Obesity poses a serious risk for some illnesses, such as diabetes, heart disease, and metabolic syndrome (28). Although BMI is a widely used measure for evaluating being overweight, it fails to consider age and gender variations into consideration. Studies have demonstrated a significant positive correlation between obesity and OAB. In a prospective study, overweight women were found to have a 5.8 times higher risk of severe OAB compared to those with normal BMI. Furthermore, patients with OAB had substantially greater BMI and WC than patients without OAB, two indications of obesity (29). Therefore, reducing body fat may help lower the risk of developing OAB. A study examining the effect of exercise on OAB symptoms in young overweight women found that after 12 weeks of exercise, these women experienced significant improvements in both weight and OAB symptoms. This suggests that exercise has a beneficial impact on weight reduction and alleviating overactive bladder symptoms (30).

The exact mechanisms underlying the relationship between obesity, overweight, and overactive bladder (OAB) are not fully understood. First, it has been suggested that being overweight increases intra-abdominal pressure, which in turn raises intra-bladder pressure, ultimately contributing to OAB (3, 31). The accumulation of visceral fat further exacerbates bladder pressure, leading to bladder neck compression and an increased risk of urinary incontinence (32). Secondly, obesity may cause hormonal changes that affect pelvic floor muscle function and consequently urethral control (33). One study found that decreased estrogen levels can lead to pelvic floor muscle atrophy, increasing the risk of urinary incontinence (34). Additionally, metabolic syndrome (MetS) has been associated with multiple risk factors, such as abdominal obesity and dyslipidemia (35, 36). Studies have shown that MetS shares similar risk factors with OAB, such as a chronic low pro-inflammatory state and insulin resistance (37). Several inflammatory mediators, including C-reactive protein (CRP) and prostaglandin E2 (PGE2), have been detected in the serum and urine of OAB patients (38, 39). MetS is associated with chronic low-grade inflammation, characterized by elevated CRP and pro-inflammatory cytokines, which may contribute to tissue fibrosis, collagen deposition, and increased bladder stiffness (37, 38). Additionally, elevated urinary PGE2 or its metabolites may serve as potential biomarkers for both OAB and MetS (40, 41). PGE2 regulates obesity-associated inflammation and insulin resistance through the EP4 receptor signaling pathway and triggers urethral contraction (42). One of the primary factors linking obesity to OAB may be visceral fat accumulation, which increases intra-abdominal pressure. This elevated pressure is transmitted to the bladder, leading to an increased bladder pressure and a higher likelihood of bladder dysfunction, including urgency and incontinence (43). Visceral fat, being metabolically active, releases various adipokines that can influence not only body fat distribution but also affect organ systems, including the urinary system. The increased intra-abdominal pressure caused by visceral fat may lead to bladder neck compression, thereby contributing to the symptoms of OAB (30, 31). Additionally, the relationship between metabolic syndrome and OAB is of particular interest. Studies have shown that components of metabolic syndrome, including abdominal obesity, dyslipidemia, and hypertension, share similar pathophysiological mechanisms with OAB, particularly involving inflammation, insulin resistance, and oxidative stress. These factors may contribute to bladder dysfunction, potentially leading to the development and exacerbation of OAB symptoms (37, 44, 45).

## 5 Strengths and limitations

This study offers notable strengths and distinct contributions. First, it offers new insights by investigating, for the first time, the association between novel anthropometric indicators and OAB in the U.S. population, identifying the waist circumference height index (WWI) as a reliable indicator of OAB risk. Second, the study’s application of multiple models and subgroup analyses, adjusted for confounding variables, demonstrated significant associations between anthropometric indicators and OAB, thereby strengthening the robustness of the findings. Third, the study’s large sample size and national representativeness strengthen the reliability and generalizability of its findings. Clinicians could use indices like WWI and RFM in routine assessments to identify individuals at higher risk for OAB, particularly those with obesity-related factors. Furthermore, these indices could guide personalized treatment plans. For instance, individuals identified as high-risk for OAB based on elevated WWI or RFM scores could be targeted for weight management programs, including dietary interventions, physical activity, and lifestyle modifications aimed at reducing visceral fat. These interventions may not only reduce the risk of OAB but could also improve other obesity-related health outcomes, such as metabolic syndrome and diabetes. We believe that integrating these novel indices into clinical workflows would enable more precise and tailored care, ultimately improving patient outcomes through early identification and targeted interventions.

Nevertheless, there are some limitations. First, the cross-sectional design of the study limits the ability to establish causal relationships. Future prospective studies are required to investigate the longitudinal impact of anthropometric indicators on OAB prevalence. Second, since all participants were from the United States, the results may not be directly applicable to populations in other countries, and caution should be taken when extrapolating the findings. New anthropometric indices require standardized measurement protocols, additional validation in diverse populations, and potential difficulties integrating them into existing clinical workflows without adequate training or resources. Therefore, further studies are necessary to assess the feasibility of integrating indices such as WWI, BRI, RFM, and ABSI into clinical workflows. These studies should also explore the predictive accuracy, cost-effectiveness, and ease of use for healthcare professionals compared with traditional measurement methods.

## 6 Conclusion

The study identified a significant positive correlation between novel body measurements and overactive bladder (OAB). Specifically, the weight-adjusted waist circumference index (WWI) and relative fat mass (RFM) showed superior diagnostic accuracy for OAB compared to traditional anthropometric measures. These findings suggest that effective management of obesity and overweight could be crucial in preventing and treating OAB. Prospective cohort studies are necessary in the future to determine the causal relationship between the two.

## Data Availability

The original contributions presented in the study are included in the article/Supplementary material, further inquiries can be directed to the corresponding author.
