# Supplementary material for: Association between novel anthropometric indices and overactive bladder: a population-based study
Source: Front Nutr. 2025 Jan 22;12:1493792. doi: 10.3389/fnut.2025.1493792 (PMC11794096; doi:10.3389/fnut.2025.1493792)
Supplement: Supplementary file 1 [file Table_1.DOCX]

**Supplementary Table 1** **Criteria for Conversion of Symptom Frequencies recorded in NHANES and OABSS Scores**

| **According to NHANES Score** | **According to OABSS Score** |
| --- | --- |
| Urge urinary incontinence frequency | Urge urinary incontinence score |
| Never | 0 |
| Less than once a month | 1 |
| A few times a month | 1 |
| A few times a week | 2 |
| Every day or night | 3 |
| Nocturia frequency | Nocturia score |
| 0 | 0 |
| 1 | 1 |
| 2 | 2 |
| 3 | 3 |
| 4 | 3 |
| 5 or more | 3 |
| When total score ≥3, the diagnosis is OAB | |

NHANES = National Health and Nutrition Examination Survey; OABSS = Overactive Bladder Symptom Score;
